# Effectiveness of exercise rehabilitation on aplastic anemia patients receiving hematopoietic stem cell transplantation: study protocol for a randomized controlled trial

**DOI:** 10.1186/s13063-024-08197-4

**Published:** 2024-06-05

**Authors:** Menghua Ye, Ting Liu, Xiaopei Mao, Xiaoxue Tan, Jin Wang, Min Xu

**Affiliations:** 1 Department of Hematology, The First Affiliated Hospital of Zhejiang Chinese Medical University (Zhejiang Provincial Hospital of Chinese Medicine), Hangzhou, China; 2 The College of Nursing, Zhejiang Chinese Medical University, Hangzhou, China; 3 Department of Orthopedics, The First Affiliated Hospital of Zhejiang Chinese Medical University (Zhejiang Provincial Hospital of Chinese Medicine), Hangzhou, China; 4 Department of Rehabilitation, The First Affiliated Hospital of Zhejiang Chinese Medical University (Zhejiang Provincial Hospital of Chinese Medicine), Hangzhou, China; 5 Department of Nursing, The First Affiliated Hospital of Zhejiang Chinese Medical University (Zhejiang Provincial Hospital of Chinese Medicine), 54 Youdian Road, Shangcheng District, Hangzhou City, Zhejiang Province People’s Republic of China

**Keywords:** Aplastic anemia, Effectiveness, Exercise rehabilitation, Hematopoietic stem cell transplantation, Quality of life, Randomized controlled trial

## Abstract

**Background:**

Although hematopoietic stem cell transplantation provides the chances of survival for aplastic anemia patients, it is also related to many treatment-related physical and psychological side effects that severely influence the quality of life. Exercise interventions have shown positive results in mixed hematology populations. The study aims to determine the effectiveness of exercise rehabilitation in improving the quality of life, fatigue, and physical function in these patients.

**Methods:**

The study will enroll a total of 82 aplastic anemia patients receiving hematopoietic stem cell transplantation. They will be randomly divided into two groups in a 1:1 ratio. The intervention group will participate in structured exercise rehabilitation (plus usual care), while control group participants will receive usual care. The exercise rehabilitation program will be performed from neutrophil and platelet engraftment until 100 days after transplantation. All outcomes will be measured at the following time points: the neutrophil and platelet engraftment (± 1day, T0), discharge from the transplantation module (± 1 day, T1), hospital discharge (± 1 day, T2), and 100 days post-transplantation (± 5 days, T3).

**Discussion:**

This study aims to assess the effectiveness of exercise rehabilitation for aplastic anemia patients receiving hematopoietic stem cell transplantation in a Chinese single center. It is particularly vital to conduct the studies in this population. Moreover, the evidence obtained from the study will provide evidence for future research and clinical practice to exercise in aplastic anemia patients.

**Trial registration:**

ChiCTR2200060762. Registered on May 2022, www.trialregister.nl/trial/7702.

**Supplementary Information:**

The online version contains supplementary material available at 10.1186/s13063-024-08197-4.

## Background

Aplastic anemia (AA) is a bone marrow failure syndrome featured by pancytopenia associated with an empty or fatty bone marrow [1]. In addition, it is a non-malignant hematologic disorder that can mostly result in anemia, bleeding, and infection [2]. The incidence of AA in Asia, especially in East Asia, is around 2-3 times higher than that in Western countries. As a large population country, the annual incidence of AA in China is approximately 7.4 per million, which should not be underestimated [3]. Currently, hematopoietic stem cell transplantation (HSCT) is an effective method to achieve a radical cure for this disease. With the maturation of transplantation techniques, expanded indications for transplantation, and improved pretreatment protocols, the number of HSCT has been on a continuous increase globally for decades [4]. As shown in the Asia Pacific Blood and Marrow Transplantation (APBMT) report, China has the second largest number of hematopoietic stem cell transplants in the Asia-Pacific region with the fastest-growing number of transplants in the region [5]. Data from the Chinese Blood and Marrow Transplantation Registry (CBMTR) demonstrated that the number of HSCT for AA (HSCT-AA) patients continues to increase. Since 2012, it has surpassed myelodysplastic syndrome (MDS) to rank third in terms of number, after acute myeloid leukemia (AML) and acute lymphoblastic leukemia (ALL) [6]. In other words, AA has become the third most common indication for HSCT. Therefore, the population of these patients deserves more clinical and research attention.

According to studies, HSCT-AA patients had a survival rate of over 85% at 10 years [7, 8], and 96% of those who survived at 25 years were in complete remission at the last follow-up visit [9]. However, it should not be overlooked that HSCT is an aggressive and demanding medical therapy. In addition, it requires conditioning regimens of high-dose chemotherapy combined with total body irradiation, followed by transfusion of donor-harvested bone marrow or peripheral blood stem cells, which can make a serious impact on the quality of life of AA patients [10]. This warns that it appears undesirable for researchers to concentrate only on the survival rate and complications of HSCT-AA patients. It is correlated with many treatment-related physical, psychological, and psychosocial side effects. This process may lead to high levels of fatigue, decreased balance, skeletal muscle strength, athletic ability, and physical functions [11]. High doses of radiotherapy cause a variety of problems for patients, including mouth ulcers, nausea and vomiting, diarrhea, dry mouth, and hair loss [12]. The closed treatment environment of the transplantation module isolates patients from the outside and creates negative emotions including helplessness, depression, loneliness, and even anxiety and depression [13]. After transplantation, patients recover from the disease, but are full of uncertainty about work, fertility, and the future, with reduced social interaction and difficulties in social return [14]. Therefore, it is of necessity to provide supportive care for HSCT-AA patients to alleviate the various problems associated with HSCT.

Currently, meta-analyses have confirmed the positive impact of exercise interventions on the physical and psychosocial functioning and quality of life of transplant patients [15, 16]. However, as evidenced by the diversity of interventions and mixed hematologic and cancer populations, most of the existing studies have methodological limitations [17]. A mixed sample including both malignant and non-malignant hematology disorders that received HSCT was selected for most studies. No subgroup analysis was performed to determine whether it was the same hematologic disease. The symptoms and treatment modalities of patients with different hematologic diseases show significant differences. For example, malignant hematologic diseases (like leukemia) have a high risk of recurrence after transplantation, and disease recurrence is the leading cause of death after transplantation [18, 19]. By contrast, benign hematologic diseases such as AA have a lower risk of recurrence after transplantation, and the leading cause of death after transplantation refers to complications including graft-versus-host disease (GVHD) [20]. These differences in clinical characteristics support the need for exercise rehabilitation intervention studies in the HSCT-AA population. Meanwhile, we believe that studies on exercise interventions for malignant hematological diseases [21], lymphoma [22], and MDS [23] are also underway. To our knowledge, there is limited published literature on exercise rehabilitation in HSCT-AA patients. It provides an opportunity to examine the effectiveness of exercise interventions in benign but severe hematologic diseases such as HSCT-AA. The study hypotheses are as follows: (1) compared to the control group, exercise rehabilitation will result in a greater improvement on QoL (primary hypothesis); (2) compared to the control group, exercise rehabilitation will result in a greater improvement in muscle strength; (3) compared to the control group, exercise rehabilitation will result in greater improvement on fatigue and hematological indicators.

## Trials design

**Fig. 1:**
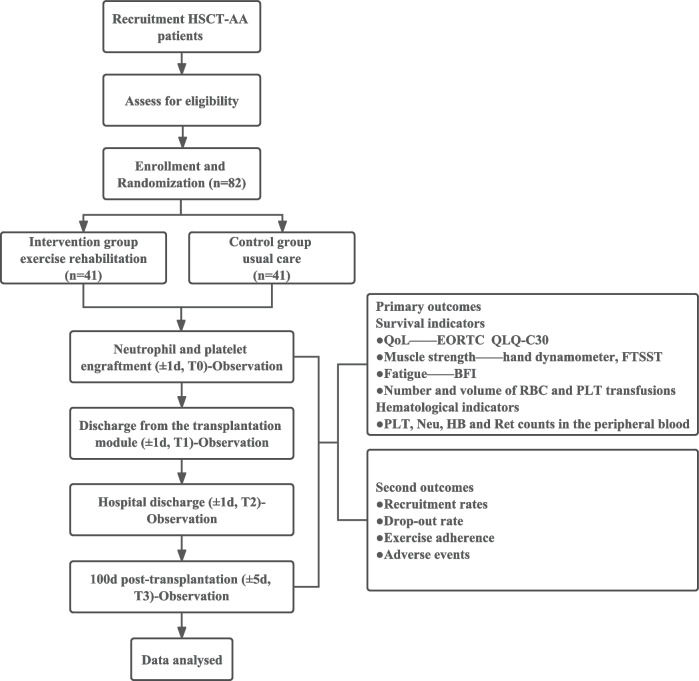
Flow chart of the study design

We will perform a randomized, controlled, assessor-blinded trial. In this trial, we will assess the effectiveness of exercise rehabilitation on HSCT-AA. The trial is registered in the Chinese Clinical Trial Registry (ChiCTR2200060762). The complete form can be accessed online at https://www.trialregister.nl/trial/7702. Patients with HSCT-AA were randomly divided into two groups: an intervention group (structured exercise rehabilitation program as the main intervention) and a control group (usual care as the main intervention). All patients in this study will start the intervention at the time of neutrophil and platelet implantation (± 1 day, T0), which lasted until 100 days post-transplantation. All outcomes were measured at the time of neutrophil and platelet engraftment (± 1 day, T0), discharge from the transplantation module (± 1 day, T1), discharge from the hospital (± 1 day, T2), and 100 days post-transplantation (± 5 days, T3). The study flow chart is displayed in Fig. 1.

## Methods

### Study setting

This study will be conducted at a single site: The First Affiliated Hospital of Zhejiang Chinese Medical University (Zhejiang Provincial Hospital of Chinese Medicine). Recruitment and outcome evaluation of this study will be conducted in the outpatient clinic and ward of the Department of Hematology of the hospital. The exercise intervention implementation part consists of three parts: hospital transplantation module, hematology ward, and out-of-hospital home environment.

### Inclusion and exclusion criteria

The patient inclusion criteria were shown below: all patients with AA referred to the hospital for consideration of first HSCT, those aged 18 years or older, and those who were willing and able to offer written informed consent.

The exclusion criteria were as follows: those with communication problems, cognitive impairment, mental disorders, and inability to cooperate in completing credible investigations; those with severe heart, lung, liver, skeletal muscle, and kidney functional changes; those complicated by other serious diseases affecting exercise rehabilitation, such as DVT, pathological fracture, and spinal cord compression; those with vision problems that interfered with the smooth performance of exercises.

### Informed consent

Participants are recruited at the Department of Hematology, The First Affiliated Hospital of Zhejiang Chinese Medical University (Zhejiang Provincial Hospital of Chinese Medicine). Hematologists will provide us with lists of AA patients who are scheduled for HSCT every day. MXP apply the inclusion and exclusion criteria to screen and subsequently recruit the patients for participation. Once patients are deemed eligible to participate, they will sign a written version of the informed consent form with voluntary informed consent, with no study procedures taking place until consent has been obtained. At T0 visit, a member of the study team will confirm eligibility.

### Intervention

The intervention group received a structured exercise rehabilitation program, which was mainly developed by our team through preliminary literature research and by inviting experts in the relevant fields (including hematologists, nurses, and rehabilitation therapists) [24]. The exercise rehabilitation was divided into 3 phases according to the different periods of the patient’s transplantation; each phase involved warm-up, formal, and finishing exercises, and was tailored according to the strength of each participant.

- Phase 1: Exercise rehabilitation in the transplantation module (neutrophil and platelet engraftment to out of the transplantation module). Considering that the patient was weak in the early post-transplantation period, exercises in this phase were mainly bed and bedside activities. The warm-up exercises were mainly lip reduction-abdominal breathing with progressive relaxation exercises (involving hand muscles, arm muscles, hip muscles, thigh muscles, calf muscles, and toe muscles), for exercising the muscles of the whole body; the aerobic exercises in formal exercises were mostly performed with bed-side bicycles; the resistance exercises were mainly bed or bedside elastic band exercises performed by the patient (involving biceps, triceps, psoas major, abdominal, gluteal and foot muscles); and the finishing exercises wrapped up with progressive relaxation exercises.
- Phase 2: Exercise rehabilitation in the hematology ward (during admission to the general ward).

Patients who were transferred from the transplant module were in better condition than before, so it was appropriate to increase the intensity of exercises and move from bed to floor. The warm-up exercise included 6 movements (including head exercise, shoulder circuit, chest expansion, waist circuit, knee circuit, and wrist and ankle exercises) to avoid injury during exercise; the aerobic exercise options for formal exercises included Baduan Jin and Tai Chi; the resistance exercise was a stretch band exercise consisting of 8 movements (more difficult than before); and the finishing exercise was a relaxing stretch of the whole body in 8 movements from head to toe.

- Phase 3: Out-of-hospital exercise rehabilitation (post-discharge to 100 days post-transplantation).

The patient continued the exercise pattern of phase 2. Meanwhile, after taking into account the patients’ return to society, exercise modalities such as cycling, jogging, brisk walking and stair climbing were added to the aerobic exercises. In addition to stretch band exercises, patients were recommended a suitable form of exercise based on the definition of 3 to 6 metabolic equivalents (MET) for moderate-intensity exercises released by the United States Department of Health and Human Services (HHS) [25].

Videos prepared by researchers were used to teach exercises of all movements. Patients could broadcast the videos on TV or mobile phones. As recommended by the experts, the warm-up exercises and finishing exercise should last for 5–10 min each time. Aerobic exercises were performed twice a week for 20–25 min. Resistance exercises were implemented thrice a week for 15–20 min, and the appropriate Thera-band elastic band was selected according to the patient’s platelet count [26]: (1) PLT < 20 × 10^9^/L: resistance exercise was not recommended; (2) 20 × 10^9^/L ≤ PLT < 30 × 10^9^/L: yellow elastic band (3.0 lbs); (3) 30 × 10^9^/ L ≤ PLT < 50 × 10^9^/L: yellow or red elastic band (3.7 lbs); (4) 50 × 10^9^/L ≤ PLT < 150 × 10^9^/L: red elastic band (3.7 lbs); and (5) PLT ≥ 150 × 10^9^/L: red or green elastic band (4.7 lbs) was recommended.

The exercise intensity was monitored using the target heart rate (THR) combined with the Rating of Perceived Exertion (RPE) Scale. To be specific, exercise intensity was targeted between 50 and 75% of heart rate (HR) reserve, in which THR = [(max HR − resting HR) × % intensity] + resting HR, where max HR = 220-age (for female) or 205-age (for male). Participants also used the RPE scale score of 12 ~ 14 points as the target exercise intensity. They followed the principle of gradual progression in the process of exercises.

Participants in the control group were randomized to usual care receiving usual care treatment according to the Chinese Expert Consensus on the Diagnosis and Treatment of Aplastic Anemia [27], including pre-HSCT workup, conditioning regimen, management of early issues, and late complications post-HSCT. In addition, access to educational materials on allogeneic bone marrow transplant was published by UpToDate [28]. Receiving health education and encouragement related to exercise, but there is no standardized program or specific requirements.

#### Criteria for discontinuing or modifying allocated interventions

This study allows patients to withdraw from the research voluntarily, and if a participant experiences an adverse reaction related to the study during the treatment process, the clinical doctor can exclude them from the study. If participants withdraw due to negative effects, the clinical doctors will assess whether they require further treatment.

#### Adherence

In addition, an exercise rehabilitation log was issued to the participants during the exercise period. The exercise log, which was required to be recorded faithfully each day, covered several aspects including type and duration of exercises, subjective exercise responses (like the assessment of pain, fatigue, emotional state, and distress), and adverse events. When no exercise was performed, the reason should be provided. The first two phases of exercise rehabilitation were supervised by the interventionists, while the third phase of out-of-hospital exercise rehabilitation was supervised by daily WeChat exercise punch cards and weekly telephone contact. The exercise rehabilitation program was from the participant’s neutrophil and platelet engraftment after transplantation (approximately 14 days post-transplantation) to 100 days post-transplantation.

### Primary and secondary outcomes

In this study, participants’ QoL was the primary outcomes, whereas participants’ muscle strength, fatigue, hematological indicators, exercise adherence, and adverse events were the secondary outcomes. All outcomes were measured at the time of neutrophil and platelet engraftment (± 1 day, T0), discharge from the transplantation module (± 1 day, T1), discharge from the hospital (± 1 day, T2), and 100 days post-transplantation (± 5 days, T3). Evaluations of T0 will be administered by the MXP enrolling the patient as part of the baseline assessment. Follow-up evaluations will be administered by TXX, who are blinded to the patient’s group allocation. All assessors will be trained by the rehabilitation therapist on the steering committee before the start of the trial, and periodic meetings between the evaluators will be established fortnightly to promote internal transparency and consolidate data collection procedures. T0, T1, and T2 measurements will be performed in the inpatient of hematology, and T3 measurements will be performed in the inpatient or outpatient clinic of hematology, depending on the patient’s follow-up results.

### Variables and measurement instruments

1. Sociodemographic and clinical variables: To characterize the samples, sociodemographic and clinical data were extracted from the participants, containing age, gender, marital status, educational level, professional/employment status, household income, duration of AA, medication, and type of treatment.
2. QoL: it was measured using the validated European Organization for Research and Treatment of Cancer Quality of Life Questionnaire-Core 30 (EORTC QLQ-C30) [29]. This questionnaire consists of 30 items covering 15 domains, which reflect the patient’s physical, role, emotional, cognitive and social functioning, a global quality of life domain, together with fatigue, nausea and vomiting, pain, dyspnea, insomnia, loss of appetite, constipation, diarrhea, and financial difficulties. All items were rated on a 4-point response scale ranging from 1 (not at all) to 4 (very much) points, except for the two items measuring general health and QoL (summarized as global QoL) that were rated on a visual analogue scale from 1 ~ 7 points. All scales were transformed to a percentile scale from 0 ~ 100 points, with the higher scores representing better functioning and global QoL, whereas the higher scores on the symptom scales indicated the higher levels of symptoms or problems.
3. Upper limb muscle strength: it was assessed by measuring the grip strength using a hand dynamometer (EH101, Camry, China) [30]. To be specific, the participant was asked to sit upright in a chair and place the feet on the floor. The shoulder of the limb to be tested was kept adducted and neutral for rotation, with the elbow being flexed at 90°, wrist extension between 0 and 30° and ulnar deviation of 0 ~ 15°. The left and right hands were alternately measured thrice each and the average value was taken as the grip strength of the participant. During the test, the participants were constantly verbally encouraged to use their maximum strength.
4. Lower limb muscle strength: it was evaluated with the Five Times Sit to Stand Test (FTSST) [31]. In brief, participants were required to sit in an armless chair at a standard height (45 cm) and tested on the duration of 5 sit-to-stand movements. In the test procedure, the participants were asked to stand upright with their upper body, with arms crossing over the chest and feet flat on the floor. Participants were asked to stand up completely without using their upper limbs and then sit down again.
5. Fatigue: it was measured with the Brief Fatigue Inventory (BFI) questionnaire [32]. The BFI questionnaire covers 10 items. The first item determines whether there has been abnormal fatigue over the last week using a “yes” or “no” option. The other items determine the current level of fatigue and the impacts of fatigue on general activity, mood, walking ability, relationships with others, and enjoyment of life. Each item is based on a 0–10-point scale, with a score of 0 representing the best state and no fatigue, whereas that of 10 indicating the worst state of fatigue. Participants were asked to mark their answers to each question based on their perceived level of fatigue, and the overall fatigue level was measured by calculating the total score of items 2 to 10 divided by 9. To be specific, a score of 0 meant no fatigue, while those of 0.1 ~ 3.9, 4 ~ 6.9, 7 ~ 9.9, and 10 were indicative of mild, moderate, severe, and very severe fatigue, separately.
6. Hematological indicators: These indicators included platelet (PLT), neutrophil (Neu), hemoglobin (HB), and reticulocyte (Ret) counts in the peripheral blood of participants.
7. Exercise adherence: it was assessed by the exercise attainment rate, which was the number of people attaining the standard/total number of people × 100%. Exercise attainment was defined as exercising at least thrice a week for 30 min or longer (or ≥ 10 min each time for a cumulative total of 30 min).
8. Adverse events: the proportion of adverse events occurring during the study period was determined.

### Participant timeline

The participant timeline is shown in Table 1.

**Table 1:** Participant timeline

	STUDY PERIOD			
	Baseline	Post-allocation		
TIMEPOINT^a^	*t*_*0*_	*t*_*1*_	*t*_*2*_	*t*_*3*_
ENROLMENT:				
Eligibility screen	X			
Informed consent	X			
*Demographics*	X			
*Medical history*	X			
INTERVENTION:				
*[Intervention group]*		X	X	X
*[Control group]*		X	X	X
ASSESSMENTS:				
*[EORTC QLQ-C30]*		X	X	X
*[hand dynamometer]*		X	X	X
*[ FTSST]*		X	X	X
*[BFI]*		X	X	X
*[Number and volume of RBC and PLT transfusions]*				X
*[Hematological indicators]*		X	X	X
*[Recruitment rate]*				X
*[Drop-out rate]*				X
*[Exercise adherence]*		X	X	X
* [Adverse events]*				X

^a^***t***_***0***_: the neutrophil and platelet engraftment (± 1day); ***t***_***1***_: discharge from the transplantation module (± 1 day); ***t***_***2***_: hospital discharge (± 1 day); ***t***_***3***_: 100 days post-transplantation (± 5 days)

### Randomization and blinding

Eligible participants will be randomly divided into two groups in a 1:1 ratio. Randomization will be conducted using an automated permuted block. The block size will not be disclosed to study staff who are enrolling patients or assigning interventions. The computer-generated randomization sequence will be kept by an independent researcher not involved in the exercise rehabilitation assessment and will be kept in a sealed envelope. Since the intervention in this study is exercise rehabilitation, the interventionists and participants will not be blinded. Participants will be informed of the intervention after completing all baseline assessments. Outcome assessors will be blinded, so unblinding will not occur. And participants will be told not to reveal the intervention they received while performing the outcome measure.

### Sample size

PASS software was employed to assess the sample size. The sample size calculations were based on previous research on physical activity for hematopoietic stem cell transplantation for QoL [33]. The study will be a parallel-group randomized controlled trial with the quality of life being the primary outcome in the study. Based on a previous study [3], the mean QoL in the control group was estimated to be 56.3 ± 17.6, and exercise rehabilitation increased the QoL in the intervention group by 12.3 relative to the control group. With an alpha risk of 0.05 (bilateral) and a beta risk of 0.20, the sample size of each group was calculated to be at least 33 people. Considering the existence of factors including shedding, the samples were expanded by 20% on the premise of guaranteeing the minimum sample size. The samples were expanded to 41 cases in each of the two groups, with a total of 82 cases.

## Oversight and monitoring

### Composition of the coordinating center and trial steering committee

The study group provided coordination and day-to-day support for the trial. The study leader, XM, supervised the design of the study and will supervise and guide the implementation of the trial. MXP is responsible for identifying potential recruits and taking consent. YMH, TL, and WJ are responsible for implementing the intervention, and TXX was responsible for data collection and management. The trial steering committee (TSC) will consist of a hematologist and a rehabilitation therapist which were independent of the study coordinating. It will meet at least every 3 months throughout the trial.

## Data management

A unique identifier will be provided for each participant who meets the inclusion criteria. An Excel file will be stored by the researcher. All data collected/analyzed will be entered into the Excel database.

### Safety/harms

Any adverse events that occur will be monitored and recorded throughout the trial. Potential minor adverse events that can be expected may be fatigue, tachycardia, dizziness, or blurred vision, which can be minimized mainly through assessment and monitoring. Based on the patient’s adverse events, a professional will assess and decide whether further treatment is required. If adverse events are reported, they will be collected and categorized according to expectedness, seriousness, severity, and potential relevance to the study. This information will then be relayed to the study’s steering committee, and the ethics committee will be informed accordingly.

### Auditing

The primary researcher will be responsible for continuous monitoring of the study and will report and discuss progress at monthly team meetings. Given that this is a single-center trial with limited sample size and low-risk interventions, a data monitoring committee has not yet been established. In the event of any adverse events, we will promptly report to the ethics committee.

### Protocol amendments

Any modifications to the study protocol will require re-approval from the respective ethics committee and will be updated on ClinicalTrials.gov.

## Statistical analysis

SPSS26.0 was adopted for statistical analysis of data. Missing data were filled in using the Last observation carried forward (LOCF) and statistical analysis was conducted following the intention-to-treat (ITT) principle. Statistical tests were carried out with two-sided tests and *P* 0.05 was considered a statistically significant difference. Gender, education level, marital status, exercise adherence, and incidence of adverse events were described as frequencies and percentages. In the meantime, age, duration of AA, time to HSCT and QoL, muscle strength, fatigue, PLT, Neu, HB, and Ret counts were described as means and standard deviations if they conformed to normal distribution, or median if they conformed to non-normal distribution. The chi-square test or *t-*test was employed to compare differences between the intervention and control groups. Taking into account the potential variations in age, gender, and education level, analysis of variance (ANOVA) was conducted to assess the development of outcomes over time. Additionally, repeated measures analysis was used to analyze scores at baseline and follow-up scores of all measures.

## Ethical considerations

The study was approved by the Ethics Committee of The First Affiliated Hospital of Zhejiang Chinese Medical University (Zhejiang Provincial Hospital of Chinese Medicine) (Ethical approval ID: 2022-KL-095-01). All participants were informed of the purpose, requirements, procedure, and study methods of the study prior to signing the written informed consent. Participants could withdraw from the study at any time without affecting their treatment. Personal data collected during the study will be kept strictly confidential and used only for this specific research project, not for any other purposes.

## Discussion

This study was aimed at evaluating the effectiveness of exercise rehabilitation in HSCT-AA patients from a Chinese single center. Currently, there is no standardized, widely accepted, and referenced exercise intervention for HSCT-AA patients, and it is particularly important to conduct such studies for this population.

The results of this study help provide scientific and practical knowledge to support the rehabilitation of HSCT-AA patients and improve their QoL. Expectedly, this study is acceptable and feasible as an important step in the field of exercise rehabilitation in benign hematological treatment and may provide useful information for health care.

### Limitations

Several limitations should be noted in this work. Firstly, HSCT-AA patients may be frail due to treatment and disease, and the patient attrition may be high after follow-up for 100 days post-transplantation. We will consider other measures to remind patients to participate in the follow-up study. Secondly, the control group was mainly provided with usual care together with exercise-related health education, which may have underestimated the intervention effect. Nevertheless, we consider that it would be unethical to include control subjects without any intervention. Finally, the white paper report mentions that HSCT patients should receive multimodal interventions, including exercise, nutrition, and return to work [34]. However, this study did not include a nutritional assessment or dietary intervention, which is considered another limitation of this exercise-focused intervention.

## Conclusions

To conclude, this study will evaluate the effectiveness of exercise rehabilitation for HSCT-AA patients. If the intervention proves to be effective, it could be used on a large scale and widely in this population, which may enhance the quality of life of patients. Moreover, the evidence obtained from this study will provide evidence for future research and clinical practice.

## Trial status

The current protocol is version 1 of May 2022. Recruitment for this study begins in February 2023. The trial status is currently in the recruitment phase. Recruitment and trial are expected to be completed by August 2024.

### Supplementary Information

Supplementary Material 1.

## Data Availability

The datasets used and/or analyzed during the current study are available from the corresponding author on reasonable request.

## Abbreviations

AA: Aplastic anemia
HSCT: Hematopoietic stem cell transplantation
GVHD: Graft-versus-host disease
QoL: Quality of life
APBMT: Asia Pacific Blood and Marrow Transplantation
CBMTR: Chinese Blood and Marrow Transplantation Registry
AML: Acute myeloid leukemia
ALL: Acute lymphoblastic leukemia
MET: Metabolic equivalents
HHS: Health and Human Services
THR: Target heart rate
RPE: Rating of Perceived Exertion
EORTC QLQ-C30: European Organization for Research and Treatment of Cancer Quality of Life Questionnaire-Core 30
FTSST: Five Times Sit to Stand Test
BFI: Brief Fatigue Inventory
RBC: Red blood cell
PLT: Platelet
Neu: Eutrophil
HB: Hemoglobin
Ret: Reticulocyte
